# The moral foundations of illusory correlation

**DOI:** 10.1371/journal.pone.0185758

**Published:** 2017-10-03

**Authors:** Javier Rodríguez-Ferreiro, Itxaso Barberia

**Affiliations:** 1 Departament de Cognició, Desenvolupament y Psicologia de la Educació, Universitat de Barcelona, Barcelona, Spain; 2 Institut de Neurociències, Universitat de Barcelona, Barcelona, Spain; Mälardalen University, SWEDEN

## Abstract

Previous research has studied the relationship between political ideology and cognitive biases, such as the tendency of conservatives to form stronger illusory correlations between negative infrequent behaviors and minority groups. We further explored these findings by studying the relation between illusory correlation and moral values. According to the moral foundations theory, liberals and conservatives differ in the relevance they concede to different moral dimensions: Care, Fairness, Loyalty, Authority, and Purity. Whereas liberals consistently endorse the Care and Fairness foundations more than the Loyalty, Authority and Purity foundations, conservatives tend to adhere to the five foundations alike. In the present study, a group of participants took part in a standard illusory correlation task in which they were presented with randomly ordered descriptions of either desirable or undesirable behaviors attributed to individuals belonging to numerically different majority and minority groups. Although the proportion of desirable and undesirable behaviors was the same in the two groups, participants attributed a higher frequency of undesirable behaviors to the minority group, thus showing the expected illusory correlation effect. Moreover, this effect was specifically associated to our participants’ scores in the Loyalty subscale of the Moral Foundations Questionnaire. These results emphasize the role of the Loyalty moral foundation in the formation of attitudes towards minorities among conservatives. Our study points out the moral system as a useful fine-grained framework to explore the complex interaction between basic cognitive processes and ideology.

## Introduction

According to the moral foundations theory [1], moral sensitivities can be structured around five dimensions: the Care foundation refers to compassion and protection from damage; the Fairness foundation relates to justice and views on how resources should be distributed; the Loyalty foundation entails commitment to the group, emphasizing the differences between in-group and out-group; the Authority foundation implies respect to hierarchy and attitudes towards obedience and leadership; the Purity foundation is related to ideas of prevention from physical or spiritual contamination. Care and Fairness are sometimes referred to as “individualizing” foundations, because they highlight individuals as the locus of moral values. On the other hand, Loyalty, Authority and Purity are considered to be “binding” foundations because they situate the focus of moral values onto groups.

Given the close relationship between moral intuitions and political inclinations, the moral foundations theory has been applied to explore the interplay between moral values and ideology, mainly focusing on the study of the liberal-conservative continuum. In many studies, liberals and conservatives have been shown to differ in the relevance they concede to different moral concerns [2–5]. Whereas liberals are known to concede more relevance to the individualizing than to the binding foundations, conservatives rate the binding foundations as more relevant than the liberals do, tending to adhere to the five foundations alike.

Differences in political perspectives have also been studied in relation to basic cognitive processes. Specifically, the influence of cognitive biases over ideology has received certain attention in the last years [6–9]. Cognitive biases are systematic errors in our reasoning that make us draw flawed conclusions and make incorrect decisions [10]. Among these phenomena, illusory correlation is of special interest from our perspective because it has been proposed as a cognitive facilitator of the development of social prejudices [11,12], which are known to be more frequent among conservatives than liberals [13–15].

Generally, illusory correlation denotes a tendency to overestimate the degree of covariation between two variables. More specifically, distinctiveness-based illusory correlation [11] refers to the predisposition to associate two relatively infrequent events in absence of real covariation. In a standard illusory correlation task, a volunteer is presented with sentences describing either positive, socially desirable, or negative, socially undesirable, behaviors performed by people from a fictitious majority or minority group. Although the ratio of positive to negative behaviors is the same in the two groups (usually 2.25:1), the participants typically overestimate the percentages of the less frequent dimension in the minority group and subjectively rate its members according to that estimation. Thus, if undesirable behaviors are the less frequent dimension, participants would end up considering members of the minority group to be less likeable.

The effects observed with this experimental task in the laboratory have been linked to social stereotype and prejudice formation in real-life environments. Human communities usually comprise different social groups varying in size, and negative behaviors are typically less frequent than positive ones. The cognitive tendency to associate the minority groups with less frequent behaviors, even in well controlled situations in which the proportions of desirable and undesirable behaviors are carefully matched, could be, at least in part, responsible for the appearance of negative group stereotypes against social minorities in real life [12].

Illusory correlation effects have already been specifically associated to political views. In this sense, Castelli and Carraro [16] showed that, compared to liberals, conservatives tend to develop stronger illusory correlation effects when negative behaviors are the infrequent ones. These effects were later replicated by the same group, and new data showed that they could be observed, not only on explicit, but also on implicit attitudes towards the minority group [17].

As political ideology is closely related to the moral system, we consider that moral intuitions might also be associated to cognitive processes like those underlying the illusory correlation effect. Moreover, we believe that, compared to the liberal-conservative dimension, moral foundations could provide a more fine-grained framework to understand the interplay between basic cognitive functions and ideology.

The way in which previous authors have determined the political views of their participants usually involves, to some extent, moral evaluation. For example, Castelli and Carraro [16] determined their participants’ political position by asking them to report their level of agreement with five different topics: reduction of immigration, medically assisted procreation, homosexual marriage, use of arms for personal defense and adoption by homosexual couples. In this sense, previous studies have observed that opinions on these and other topics are specifically predicted by differential endorsement of specific moral foundations. For example, Koleva et al. [18] observed that Purity is the best predictor of attitudes towards same-sex marriage whereas Care is the strongest predictor of support for gun control.

Hence, we believe that, by approaching the relationship between ideology and propensity to develop illusory correlations from the perspective of the moral foundations theory, we can more precisely determine which specific aspects of being a conservative or being a liberal are related to the tendency to develop illusory correlations. That is, if conservatives differ from liberals in the importance they give to different moral foundations, then it is possible that only some of these foundations are significantly related with the illusory correlation effect. Our initial hypothesis is that endorsement of binding moral dimensions, typically more strongly present in conservatives, will be positively related to illusory correlation effects, whereas intuitions associated to individualizing moral foundations will not. As illusory correlation is closely related to the generation and maintenance of prejudices and social stereotypes, we propose that scores in the Loyalty foundation, which focuses on the distinction between in-group and out-group members, will be the most important dimension of this association. Loyalty-related moral concerns imply beliefs that members of one’s own group should be treated more favorably than members of other groups. For instance, those who report higher Loyalty concerns tend to favor in-group members, such as relatives, friends, neighbors or people from their same sports team or country. From our perspective, the theoretical relevance of Loyalty in relation to in-group preference situates this foundation as a straightforward candidate to be in the core of the association between conservatism and illusory correlation.

## Methods

### Participants

Data was gathered in two phases. In the first stage, an initial group of 132 volunteers took part in the experiment. After preliminary analyses and following the reviewers advice on a previous version of the manuscript, we increased the sample size up to 263 participants. They were all students at the University of Barcelona who took part in the study in exchange of course credits. Twenty-three students were excluded because they did not complete the three tasks, so the final sample consisted of 240 volunteers (80% females, mean age 23.8, SD 4.47). The study was conducted in Spanish. Data were gathered and analyzed anonymously. Prior to their participation in the study the participants were naïve to the topics of illusory correlation and moral foundations. The study protocols were approved by the university’s ethics committee (Comissió de Bioètica de la Universitat de Barcelona, CBUB) and written (on-line) informed consent was obtained from all the volunteers prior to their participation in the study.

### Procedure

The study consisted of three phases, the illusory correlation task, the moral foundations questionnaire and a political identity survey, which were conducted by the volunteers individually in three separate online sessions presented one week apart.

In the illusory correlation task [11] participants were explained that they were going to be shown faces of people living in two different districts. Each face would be presented in the computer screen along with a sentence describing a behavior performed by that person. Volunteers were instructed to read the sentences carefully because they would be later asked questions about the people in each district and their behaviors. The sentences could describe either desirable (e.g. “… visited a sick friend in the hospital”) or undesirable (e.g. “… cheated on an exam”) behaviors, and most of them were adapted and translated into Spanish from [19]. We used faces showing neutral emotions from the Karolinska Directed Emotional Faces image set [20]. The task consisted of 39 trials. In each trial the face appeared in the center of the screen with the name of the district, A or B, and the sentence describing the behavior underneath. In order to facilitate the discrimination between the two districts we made use of two simple stock drawings of skylines and two different background colors (green or blue). Depending on the district, one of the two skylines was added to the left of the face and one of the two background colors was selected accordingly. Two thirds of the faces presented, 26 trials, corresponded to one of the districts, the majority group, and the other third, 13 trials, corresponded to the other district, the minority group. The distribution of the specific sentences was randomized across participants and districts but a 2.25 to 1 ratio between sentences describing positive and negative behaviors in each group was always maintained. All in all, 18 positive and 8 negative sentences appeared in the majority group, whereas 9 positive and 4 negative sentences appeared in the minority group. The correspondence between the majority and minority groups and the skyline images, background colors, district names and face photos were randomized across participants. The participants controlled the pace of the task by pressing the spacebar to change from one trial to another.

After the participants had run through all the trials they were asked to respond to two different types of questions: evaluative ratings and frequency estimates. First the volunteers had to rate in a 1 to 7 Likert-like scale to which extent they thought different adjectives [19] generally applied to the neighbors in each district. There were four positive (i.e. “honest”, “intelligent”, “popular” and “helpful”) and four negative (i.e. “irresponsible”, “lazy”, “unpleasant” and “miserable”) adjectives for each district. Then in a final question the volunteers had to express how many of the total amount of residents in each district presented negative behaviors (e.g. “You have been presented with 26 sentences corresponding to people from neighborhood A. Could you estimate how many of those 26 sentences referred to disagreeable attitudes or behaviors?”).

In the second phase of the study the participants had to respond to a Spanish-translated version of the MFQ30 test [21], which assesses the relevance one concedes to five different moral dimensions. The test consists of 30 items, plus two control questions, with six items corresponding to each foundation. In the first half of the questions participants are asked to respond how important a given consideration is when deciding if something is right or wrong (e.g. “Whether or not someone suffered emotionally”). In the second half of the questions volunteers are asked to indicate their degree of agreement with given morally relevant statements (e.g. “Compassion for those who are suffering is the most crucial virtue”). Responses are registered using a six-point Likert-like scale with higher responses denoting greater moral relevance. Cronbach’s α for the different subscales of the test in our sample were rather low: .42 for Care, .58 for Fairness, .67 for Loyalty, .64 for Authority and .62 for Purity. Reliability analyses of the five subscales showed that eliminating item 28 from the Care subscale (“It can never be right to kill a human being”) increased its alpha value up to .57. Similarly, removal of item 29, corresponding to the Fairness subscale (“I think it’s morally wrong that rich children inherit a lot of money while poor children inherit nothing.”), increased its alpha value up to .67, whereas deleting item 26, corresponding to the Authority subscale (“Men and women each have different roles to play in society”) improved its alpha value up to .69. Loyalty and Purity subscales did not benefit from removal of any specific item. Results reported in the following section were obtained analyzing the improved versions of the subscales, however, the pattern of results is similar to that obtained with the full versions.

In the third phase, we assessed our participant’s political inclinations and gathered sociodemographic data. We constructed a 24-item Political Identity questionnaire inspired by the website “The Political Compass” (http://www.politicalcompass.org). For the sake of coherence with the MFQ30 we used a six-point scale, where six indicated conservative views, instead of the four-point scale used in the original website. The questions covered different topics including civil liberties, immigration, gender roles, abortion, adoption by same-sex couples, religion at schools, private health care and education services, tax distribution or free market (the full scale is provided as supporting information, S1 File). Reliability estimates for the questionnaire were acceptable (α = .85). Questions regarding age and gender were also included in the final questionnaire.

## Results

The results of the study are available through the Universitat de Barcelona Digital Repository and were analyzed by means of SPSS 23. We first analyzed the results of the illusory correlation task. We measured our participants’ attitudes, evaluative ratings, towards the majority and minority groups subtracting the mean ratings corresponding to the negative adjectives from the mean ratings obtained in response to the positive ones. In our final measure, thus, differentials above zero indicate overall positive evaluations whereas differentials below zero show overall negative evaluations (a summary of our results is provided in Table 1). The other dependent variable in this task was the perceived frequencies of negative behaviors in each group, which were converted to percentages for the analysis. Kolmogorov-Smirnov tests showed that these dependent variables were not distributed normally (ps<=.03) so we used non-parametric tests to conduct the following analyses. The comparison between the subjective differentials corresponding to the minority and majority groups showed that attitudes towards the majority group were more positive than those corresponding to the minority group (z = -4.846, p<.001, r = 0.22). Besides, participants reported significantly higher percentages of negative behaviors in the minority compared to the majority group (z = -5.68, p<.001, r = 0.26).

**Table 1:** Summary of the results obtained in the illusory correlation task.

	Majority	Minority	Difference
	mean(SD)	mean(SD)	mean(SD)
Subjective Ratings^a^			
Positive	4.64(1.1)	4.16(1.00)	
Negative	3.06(1.14)	3.48(1.21)	
Difference	1.58(1.73)	0.68(1.65)	0.9(2.82)
Frequency Estimates^b^			
Negative	43.64(16.15)	52.05(15.18)	8.41(22.58)

^a^ scores in 1–7 scale;

^b^ percentages

Second, we analyzed the results of the MFQ30 questionnaire and we checked whether the association between moral conceptions and political ideology was replicated in our sample. The participants scored relatively high in the two individualizing foundations of the MFQ30 (Care: mean = 4.72, SD = 0.61; Fairness: mean = 5.07. SD = 0.55), compared to the lower scores observed in response to the three binding foundations (Loyalty: mean = 3.25, SD = 0.72; Authority: mean = 3.31, SD = 0.78; Purity: mean = 2.58, SD = 0.7). A correlation analysis showed that the three binding foundations were significantly associated with the scores in the political identity questionnaire (see Table 2).

**Table 2:** Correlation indexes (ρ) between moral foundations and political identity questionnaires.

	Care	Fairness	Loyalty	Authority	Purity
PI	.023	-.106	.425***	.329***	.411***
Care		.481***	.300***	.283***	.176**
Fairness			.210**	.235***	.102
Loyalty				.573***	.510***
Authority					.483***

* p<.05;

** p<.01;

*** p<.001

Indiv.: Individualizing moral foundations average; Bind.: Binding moral foundations average; PI: Political Identity

Finally, we explored the relation between the results observed in the illusory correlation task and the scores obtained in the moral foundations questionnaire. We started by summarizing the evaluative ratings of our participants in the illusory correlation task. We calculated a new variable subtracting the differential adjective ratings corresponding to the minority group from those of the majority group (see the Difference column in Table 1). Values over zero in this measure indicate overall preference for the majority group, whereas values below zero indicate overall preference for the minority group. We also summarized the frequency estimates subtracting the percentages of undesirable behaviors attributed to the majority group from those of the minority group. Values denote the percentage of negative behaviors attributed to the minority group compared to the majority group. A correlation analysis including the scores in the moral foundations and the frequency and evaluative judgments of the illusory correlation task showed no significant relation between any specific individualizing moral foundations and the illusory correlation effects (see Table 3). On the contrary, we observed significant associations between illusory correlation effects and binding foundations. Specifically, Loyalty positively correlated with the frequency estimates (p = .003), an effect that survived Bonferroni correction for multiple comparisons. Finally, the results of the political identity questionnaire did not correlate with the frequency or subjective ratings obtained in the illusory correlation task.

**Table 3:** Correlation indexes (ρ) between Illusory correlation estimates and scores in the moral foundations questionnaire.

	Care	Fairness	Loyalty	Authority	Purity	PI
Subj.	.034	.001	.111	.120	.075	.011
Freq.	.003	-.013	.192**	.104	.08	.099

* p<.05;

** p<.01

Subj.: Differential of the subjective Ratings; Freq.: Differential of the frequency estimates; PI: Political Identity

Additionally, we conducted two separate regression analyses in order to state whether any of the specific moral dimensions significantly predicted the evaluative and frequency estimates provided in the illusory correlation task. The first regression analysis showed that none of the five moral foundations appeared to be a significant predictor of the subjective preference for the majority group (all ps>.287). In contrast, the second analysis pointed out Loyalty as the only significant predictor of differential frequency estimations (β = .220, t = 2.622, p = .009). Participants who endorsed the Loyalty foundation to a greater extent estimated larger percentages of negative behaviors in the minority compared to the majority group. None of the other moral dimensions showed a significant effect (ps>.565) over this dependent variable. Finally, a regression analysis including only Loyalty and political identity scores as predictors showed significant effects of the former (Loyalty: β = .183, t = 2.603, p = .01) but not the latter (political identity: β = .021, t = 0.298, p = .766) over frequency estimations.

## Discussion

The aim of this study was to explore the relationship between basic cognitive processes and moral intuitions through the assessment of the possible association between illusory correlation effects and endorsement of different moral dimensions. The results of our illusory correlation task replicated the classic findings [11,12], as the participants developed more negative attitudes towards the members of the minority group and tended to associate it to higher frequencies of negative behaviors, in spite of equal proportions of desirable-to-undesirable behaviors in the majority and minority groups. On the other hand, the second phase of the study confirmed the relation between moral foundations and ideology found in previous studies [2]. As anticipated, more conservative participants rated binding foundations as more relevant than more liberal ones, hence presenting less difference between those two moral dimension clusters.

Conjoint analyses of the results of the two first phases of the study pointed out a significant relation between some of the illusory correlation effects and specific binding foundations whereas no significant association between individualizing moral foundations and the cognitive bias were observed. Crucially, frequency estimates from the illusory correlation task were significantly predicted by Loyalty scores.

Previous studies have shown that, compared to liberals, conservatives display more pronounced selective attention for negative stimuli [22–24]. This tendency has been proposed as a possible cause of the predisposition of conservatives to develop stronger associations between minorities and negative behaviors in the illusory correlation task [16]. However, it remains unclear why this negativity bias specifically affects the minority group. If conservatives tend to pay more attention to negative stimuli then they should equally focus their attention at undesirable behaviors in the two groups. This would lead them to produce more negative estimates in relation to both groups, instead of generating a stronger association between the minority group and undesirable behaviors. Hence, a mechanism that explains the differential attribution of negative estimates to the minority is still needed.

The specific association between the Loyalty moral foundation and illusory correlation observed in the frequency estimates of our participants offers a new perspective on this issue. Loyalty-related moral concerns imply a preference for in-group over out-group members. Previous studies exploring the influence of group membership on the formation of the illusory correlation effect have shown that participants who are assigned to one of the groups prior to the task tend to favor their in-group in the frequency and, to a lesser extent, in the evaluative judgments [25,26]. We hypothesize that the degree of identification with each of the two groups could play a critical role in the relation between illusory correlation and ideology. Conservatives, who typically obtain higher Loyalty scores than liberals, have been shown to display less need for uniqueness [27], what has been directly associated to an increased tendency to endorse the majority viewpoints [28]. In our study, participants who more strongly endorsed Loyalty-related values displayed a tendency to overestimate the differences between percentages of negative behaviors in the minority, compared to the majority, group. Following previous research on the in-group bias [25,26] a tendency to over-identify with the majority group by volunteers with high Loyalty scores could potentially explain this observation. This interpretation is tentative but further studies could be conducted to explore its plausibility. From this perspective, we anticipate that when presented with situations in which negative behaviors are more frequent and, therefore, an illusory correlation favoring the minority is expected, the conservatives’ identification with the majority should moderate the effect. That is, their motivation to favor the majority group would make, in this case, the illusory correlation weaker.

In contrast with the results observed in previous studies [16,17], scores obtained in the political identity questionnaire did not correlate with illusory correlation effects. A potential explanation to these differences could be related to the way in which political ideology has been measured in the different studies. Castelli and Carraro [16] based their assessment on social issues which are usually rejected by conservatives, such as gay marriage or medically assisted procreation. As already noted by these authors (pp. 1015), in that kind of measure conservatism is confounded with avoidance motivations which could be the real source of the observed effects. In this sense, the inclusion of topics typically favored by conservatives such as private health care or tax reduction in our political identity questionnaire makes it difficult to directly compare the two studies. Furthermore, the fact that issues like the ones we just mentioned refer to economic, and not social, conservatism also adds to the idea that our PI test and the mainly social questions used by Castelli and Carraro [16] might be qualitatively different measures. Although usually associated with each other, social and economic conservatism are known to be differentially related to specific attitudes [29] or moral preferences [30]. The inclusion of economic issues in our questionnaire might have undermined its sensitivity to assess social conservatism, what could explain the lack of effects observed in our data in comparison to previous studies.

In sum, our results contribute to the understanding of which specific components of the conservative values are related to the sensitivity to develop illusory correlations, which could be taking part in the development of prejudices against social minorities. As predicted by our initial hypothesis, the Loyalty moral foundation played a crucial role in this association, pointing out the moral system as a useful framework to explore the complex interaction between basic cognitive processes and political ideology, and providing a more fine-grained tool than the liberal-conservative distinction.

The correlational nature of our study does not enable causal inferences so it is not possible for us to establish the direction of the relationship between cognitive processes and moral intuitions. However, we reckon this association might be driven by complex bidirectional influences which could be explored by means of further studies implementing experimental manipulations of these variables. For instance, if endorsement of Loyalty influences illusory correlation, then creating contexts that emphasize Loyalty-related concerns should enhance the illusory correlation effects. Additionally, longitudinal studies measuring both moral inclinations and cognitive effects over time could shed some light on the developmental course of this association.

A potential limitation of this study is that our participants were quite homogeneous regarding their moral opinions, generally reflecting high endorsement of individualizing foundations and intermediate endorsement of binding foundations. New studies conducted with more heterogeneous samples, and thus, offering more variability in relation to moral intuitions could yield stronger results, and help clarifying the exact role of the different foundations in the relationship between moral concerns and basic cognitive processes like illusory correlation.

## Supporting information

S1 File Political identity questionnaire items. (DOCX)
