# Supplementary material for: The moral foundations of illusory correlation
Source: PLoS One. 2017 Oct 3;12(10):e0185758. doi: 10.1371/journal.pone.0185758 (PMC5626483; doi:10.1371/journal.pone.0185758)
Supplement: S1 File — (DOCX) [file pone.0185758.s001.docx]

**S1 Supporting Information. Political identity questionnaire items**

| Spanish items | English translation |
| --- | --- |
| Cada uno debería encargarse de su salud contratando su propio seguro médico privado | Everyone should take care of their health taking their own private health insurance |
| Por el bien de los niños, una pareja del mismo sexo no debería poder adoptar | ^1^For the good of children, a same-sex couple shouldn't be allowed to adopt |
| Siempre defenderé mi país, aunque no tenga toda la razón | ^2^I'd always support my country, whether it was right or wrong. |
| Si los más ricos pagan demasiados impuestos la economía no funciona adecuadamente | ^1^If the rich are too highly taxed the economy does not work well |
| El aborto, cuando la vida de la madre no está en peligro, debería ser ilegal | ^2^Abortion, when the woman's life is not threatened, should always be illegal. |
| Aunque no elegimos el lugar en que nacemos tiene sentido sentirse orgulloso de ello | ^1^Although we don't choose the place where we are born it makes sense to be proud of it |
| En el sistema judicial, además de la rehabilitación, el castigo es importante para dar ejemplo | ^1^In criminal justice, punishment is important along rehabilitation |
| La nacionalidad es más importante en la identidad propia que otros factores como la clase social | ^1^Nationality is more important to identity than other factors like social class |
| La competencia entre distintas empresas privadas en un mismo sector es mejor para la economía que una sola empresa pública | Competition between different private companies in the same sector is better for economy than only one public corporation |
| Los habitantes de mi país tienen mejores cualidades que los de otros | ^2^My country has many superior qualities, compared with others |
| Las empresas privadas gestionan mejor recursos como el agua o la electricidad que las empresas públicas | Private corporations manage resources like water or electricity better than public companies |
| Es importante que en la escuela se impartan valores religiosos | ^2^It is important that my child's school instills religious values |
| La nación es una base legítima para construir un estado propio | A nation is a legitimate base to build a state |
| Las personas con más recursos deben poder enviar a sus hijos a escuelas privadas de mayor calidad | ^1^People with more resources should be able to send their kids to higher quality private schools |
| Es importante mantener las tradiciones de nuestros antepasados | It is important to maintain our ancestors traditions |
| La única responsabilidad social de una empresa debe ser conseguir beneficios para sus accionistas | ^2^The only social responsibility of a company should be to deliver a profit to its shareholders |
| La tierra natal es un importante factor definitorio de la persona | The place of birth is an important defining factor of a person |
| Aunque las mujeres tengan una carrera profesional, su principal deber es atender su hogar | ^2^Mothers may have careers, but their first duty is to be homemakers |
| Los hijos de padres inmigrantes nunca llegan a adaptarse plenamente al país | ^2^First-generation immigrants can never be fully integrated within their new country. |
| Que las grandes compañías privadas tengan éxito acaba siendo bueno para toda la sociedad | ^2^What's good for the most successful corporations is always, ultimately, good for all of us. |
| Es importante preservar los rasgos identitarios de nuestra nación | It is important to keep our nation identity features |
| La amenaza terrorista justifica el recorte de ciertas libertades civiles | ^1^Terrorist threat justifies a cut in certain civil liberties |
| No se deberían financiar mediante impuestos museos o teatros que no sean rentables por sí mismos | ^2^Taxpayers should not be expected to prop up any theatres or museums that cannot survive on a commercial basis |
| La misión fundamental de la escolarización es preparar a los estudiantes para encontrar trabajo | ^2^The prime function of schooling should be to equip the future generation to find jobs. |

^1^Adapted from The Political Compass

^2^Translated from The Political Compass
